# STRENGTHENING FAMILY CAREGIVER SUPPORTS: QUALITATIVE FINDINGS FROM THE ADULT DAY SERVICES PLUS PROGRAM TRIAL

**DOI:** 10.1093/geroni/igae098.3417

**Published:** 2024-12-31

**Authors:** Dionne Bailey, Quinton Cotton, Katherine Marx, Laura Gitlin, Joseph Gaugler

**Affiliations:** University of Minnesota, Minneapolis, Minnesota, United States; University of Minnesota, Minneapolis, Minnesota, United States; Johns Hopkins University, Baltimore, Maryland, United States; Drexel University, Philadelphia, Pennsylvania, United States; University of Minnesota, Minneapolis, Minnesota, United States

## Abstract

Home and community-based services, such as adult day services (ADS), that support people with dementia (PLWD) and their family caregivers frequently fail to meet the full range of dementia care and management needs. There is insufficient data on implementation mechanisms that achieve desirable results among existing interventions targeting dementia family caregivers. In a pragmatic trial, we evaluated the feasibility and acceptability of integrating a caregiver-specific intervention [ADS Plus] delivered through adult day services. Post-intervention, we conducted a directed content analysis using the Consolidated Framework for Implementation Research, drawing from semi-structured interviews with dementia family caregivers (n=15) and ADS site staff (n=14). Our findings demonstrate that key aspects of ADS Plus supported delivery and uptake: intervention adaptability, personalization, and structure (innovation); responsive to external changes and intervention marketability (outer domain); aligned goals and familiarity (inner setting); involvement of research staff, connections among practitioners, and meeting caregiver needs (individual domain), and; understanding caregivers needs and addressing staff capacity to take action (implementation process). ADS Plus offers a viable community-based solution for supporting dementia family caregivers. Future studies can assess fidelity across diverse implementation contexts to ensure greater transferability of findings.

